# Qualitative Evaluation of the Five-Year ‘Red Collar’ Campaign to End Inhumane Culling of Dogs as a Method of Rabies Control

**DOI:** 10.3390/vetsci5010018

**Published:** 2018-02-06

**Authors:** Elly Hiby, Lou Tasker

**Affiliations:** 1 Independent Consultant, Cambridge CB23 7EJ, UK; ellyhiby@gmail.com; 2 Independent Consultant, Derbyshire DE7 4QQ, UK

**Keywords:** rabies, domestic dog, canine, humane rabies control, mass dog vaccination, culling, campaign evaluation

## Abstract

Dog-mediated human rabies can be eliminated through mass dog vaccination. Despite leading authorities in human and animal health uniting to advance effective and humane rabies control, some governments resort to lethal methods, which are unethical, often inhumane and ineffective. To end the inhumane culling of dogs in response to rabies, World Animal Protection launched ‘Red Collar’; a five-year campaign (2011–2016) that worked with governments to promote the implementation of mass dog vaccination for rabies control. We present the findings from a qualitative evaluation of ‘Red Collar’, conducted both regionally and with national focus on Bangladesh, China, Indonesia, the Philippines and Zanzibar, Tanzania. Through semi-structured interviews and written contributions from stakeholders (*n* = 54), we compared perceptions of changes with stated campaign goals to capture recommendations for future work. The campaign successfully generated momentum for implementation of mass dog vaccination by targeted governments. Lessons learned were established: Value of a consistent animal welfare ‘voice’; the need to explore the motivations behind culling; the need to capacity build; time required for the ‘ripple effect’ to inspire humane control in other countries; importance of monitoring and evaluation of indicators; time and effort required for exit strategies and prior preparation for a robust response to culling.

## 1. Introduction

The rabies virus is one of the oldest recognized and deadliest known diseases, naturally infecting mammals including humans [1]. Human rabies is vaccine preventable, but almost always fatal following the onset of clinical signs. Despite the known lethal consequences of untreated infections, human deaths from rabies are underreported [2,3], and in many countries the disease is neglected [4]. Dog-mediated transmission of the virus is the most common route of human infection, with 99% of human cases occurring via the bite of an infected domestic dog (*Canis lupus familiaris*) [5]. This burden is substantial in countries where rabies is endemic and is estimated to result in 59,000 human deaths worldwide each year [2], mostly in Asia and Africa [6].

Domestic dogs are globally distributed and prevalent [7]. Their proliferation is inextricably linked to humans—where there are humans there are dogs—the size of the two populations positively correlate [8] and the two share an unparalleled inter-species relationship [9]. Through this close association, dogs contribute to zoonotic infections in humans—rabies being the most feared—and in response, governments frequently employ methods to reduce dog numbers including culling. However, reduction in dog density has no evidence-based impact on the control of rabies [10,11,12]. The methods used for culling are often inhumane, causing pain and distress to dogs (the World Organisation for Animal Health OIE, provides a list of unacceptable methods in their stray dog population control standard [13]). Indeed, inhumane culling is frequently unacceptable to local communities who, despite common misconceptions, consider themselves the owners or carers of the dogs, including those that roam freely [14]. Furthermore, proof-of-concept projects demonstrate that canine rabies can feasibly be eliminated through the vaccination of at least 70% of dogs during annual campaigns [4,15]. This approach is jointly advocated by leading human and animal health authorities (World Health Organization WHO, OIE and Food and Agriculture Organization of the United Nations FAO), who have united to eliminate dog-mediated human rabies deaths by 2030 [16]. 

The ‘Red Collar’ global campaign was conducted by World Animal Protection (formerly World Society for the Protection of Animals WSPA) over five years. Through advocacy and demonstration, using pilot projects in key countries, it aimed to generate momentum for governments to adopt humane, effective and sustainable control of dog-mediated rabies, founded in mass dog vaccination without culling (euthanasia of suspect rabid dogs using humane methods was encouraged). In short, it intended to remove rabies as an excuse for the inhumane culling of dogs worldwide [17]. To explore the effectiveness of the campaign in meeting this goal, an evaluation was commissioned. Here we report the findings of a qualitative assessment, outlining lessons learned, with applications to future campaigns aiming to promote dog welfare globally. We make recommendations with a wider audience in mind; those sharing a common goal of humanely and effectively reducing the burden of dog-mediated rabies on human communities.

## 2. Methods

Following the conclusion of the ‘Red Collar’ campaign (World Rabies Day 2016, 28th September), an evaluation was commissioned by World Animal Protection, and subsequently designed and undertaken by two external evaluators (E.H. and L.T.). The evaluation process spanned November 2016 to April 2017 and reviewed all regional activities that occurred between World Rabies Day 2011 to 2016 in the World Animal Protection Asia-Pacific and Africa divisions. The collaboration with the Association of Southeast Asian Nations (ASEAN), the pan-regional body that promotes cooperation between ten Member States, to progress the ASEAN rabies elimination strategy [18] was included for evaluation. The campaign also opted to work with governments located in Bangladesh, China, Indonesia, the Philippines and Zanzibar; countries or districts with a history of inhumane culling for rabies control. 

The qualitative evaluation framework and methodology was designed to follow the campaign structure and deduce common themes [19] in campaign achievements, challenges and attributions [20]. Semi-structured interviews were conducted with stakeholder representatives (*n* = 50). In-person interviews (*n* = 25) were recorded during two country visits (Bangladesh, December 2016; Zanzibar, January 2017); the remainder were conducted over Skype (*n* = 25), and four written submissions were also included for analysis. Participants included regional campaign staff and external consultants, collaborators from nongovernmental animal welfare organizations (NGOs), intergovernmental organizations (IGOs), and official in-country representatives from human and animal health. Table 1 provides a breakdown of stakeholder type and location. Participants were selected from individuals and organizations named in campaign plans and reports, those indicated as relevant by campaign staff, and ‘snowballing’ from interviewees. Key elements of the participants’ accounts and information drawn from written material, were organized into themes to compare the perceived results of the campaign with the campaign goals and theoretical predictions. Wherever published material on policy or practice was available, this was used to corroborate participants’ accounts. This was then further developed into key learning points and recommendations for the development of future campaigns.

This evaluation formed part of an end-of-campaign review, as opposed to a standalone research initiative. Therefore, it was not submitted for external ethical review, as this was not standard practice for the commissioning NGO. However, the authors, together with campaign staff, agreed to apply the relevant ethical principles outlined in the Declaration of Helsinki [21], with the exception of prospective ethical review. The external evaluators entered into a consent dialogue with participants at the start of interview, providing information relating to the aims, methods, reporting process and the intended use by the commissioning organization. Participants were informed of their right to voluntary withdrawal at any point during interview without penalty, anonymity was offered, and verbal informed consent was sought and freely given.

## 3. Results

Using participatory approaches, we captured the perception of stakeholder representatives (*n* = 54) of changes that had occurred during the five-year period of the campaign, and where it was reasonable to attribute at least some of these changes to the ‘Red Collar’ campaign. Achievements were evident in all countries/districts that had been specifically targeted for the campaign. Deeper exploration of the attribution of the campaign to these changes exposed many learning points covering both positive aspects of the campaign and potential weaknesses. The following highlights the main lessons learned and provides recommendations for future campaigns.

### 3.1. Changes in Policy and Practice

In Bangladesh and Indonesia, the policy in central government changed to humane control founded on mass dog vaccination. However, challenges with implementing these policies at a local level were still evident. Pilot areas within China were practising humane control, but changes in national policy were still in progress. In Zanzibar, humane rabies control was practised throughout the island, was embedded in government policy, and was due to be enshrined in law; however, this was following prolonged supportive engagement with World Animal Protection far exceeding the five years of the ‘Red Collar’ campaign. The campaign’s approach of combining advocacy to build political will for humane control with demonstration and capacity-building through pilot projects is effective but appears to require engagement beyond the five-year campaign timeframe to establish sustainable change in both policy and practice.

### 3.2. Compassionate Animal Welfare ‘Voice’

The consistent messaging emphasizing the need for compassion towards animals throughout rabies control provided by ‘Red Collar’ was valued by stakeholders and noted as being absent or fragmented prior to the campaign. The language of humane rabies control is now increasingly common and ‘mainstream’ (e.g., ‘*The vaccinated dog is the soldier in the war against rabies’* [4] (p. 4)). We recommend future campaigns establishing the relevance of animal welfare within other fields, such as human health, should follow similar strong and compassionate messaging.

### 3.3. Assumption that Culling Was Rabies Motivated

A founding assumption of ‘Red Collar’ was that inhumane culling was principally motivated by a desire to control rabies. This appears to have been true in some cases, but there were additional concerns about the density of roaming dogs and dog bites driving culling. Culling of dogs in the Philippines using exhaust fumes (‘Tambutso’ gassing), at least partially motivated by rabies control, appeared to have halted. However, this had also been a target of campaigns by other organizations and had benefited from World Animal Protection support for developing humane dog population management, which appears to have contributed to ending culling. The cessation of culling in Zanzibar also appears to have been facilitated through World Animal Protection support of both vaccination and humane dog population management over a long period of engagement (exceeding ten years). In other locations, the effectiveness of vaccination for preventing rabies transmission was well established, but inhumane culling was applied in addition, apparently motivated by a desire to increase vaccination coverage by targeting unvaccinated dogs with culling instead of more intensive vaccination efforts. More detailed exploration of the drivers of culling at the outset may have been beneficial; a humane alternative must address the same motivations completely, without leaving significant residual concerns that may continue to drive culling.

### 3.4. Capacity Building 

Implementing mass dog vaccination was poorly understood before the campaign in most target countries. Stakeholders reported that they valued the support for capacity-building in-country (e.g., effective training in the humane handling and vaccination of dogs) and that this was critical for changing to a humane approach and sustaining this beyond the end of the campaign. Capacity-building was provided either directly by World Animal Protection or delivered in partnership with other embedded agencies (e.g., FAO). Future campaigns should establish capacity-building more clearly as an essential step in their theory of change (‘theory of change’ is a concept associated with campaign logic that maps the pathway of steps leading to the desired impact, e.g., [22]), as a fundamental building block for change to sustained humane practice. 

### 3.5. Time Required for ‘Ripple Effect’ 

A significant theory of change underlying the campaign was that effective pilots of humane rabies control would inspire other countries to change to more humane approaches. This would create a ‘ripple effect’, cascading through replication, supported by regional bodies such as ASEAN; the ASEAN had adopted a Rabies Elimination Strategy founded on humane methods in 2015, providing influential support to Member States [18]. This theory of change has not been disproven; however, the time required to progress pilots to a stage of being robustly evidence-based and inspirational, prompting replication in other countries, is in excess of five years. There is some evidence that the ripple effect is in action, with Vietnam’s recent launch of a humane rabies control program for 2017–2021 [23]. Although this cannot be solely attributed to ‘Red Collar’, the level of engagement with Vietnam through ‘Red Collar’ activities suggest there was a contribution. Furthermore, Cambodia, an ASEAN Member State, has recently committed to eliminate rabies by 2025, in line with the ASEAN Rabies Elimination Strategy [24]. 

### 3.6. Monitoring and Evaluation

The term ‘effective’ appeared frequently in ‘Red Collar’ messaging regarding humane rabies control. There is a large body of evidence that mass dog vaccination is an effective method of controlling dog-mediated human rabies [15,25,26,27]. However, the pilot projects supported by ‘Red Collar’ did not benefit from robust data collection and analysis, and so did not contribute to this evidence base. They did, however, provide working examples of how to implement humane rabies control in practice (e.g., [4]). Future campaigns need to establish sound monitoring and evaluation plans, taking note of the indicators that will influence target stakeholders. This requires investment in scientific support to ensure robustness of data and analysis, either in-house or accessing external research capacity (e.g., from an experienced University research group or organization).

### 3.7. Exit Strategies

Establishing a sustained change to humane rabies control requires a change in in-country policy, capacity, practice and funding [16]. The timeline for ‘Red Collar’ required exit from countries that had been supported with World Animal Protection funding and training within one to two years of a pilot commencing. This time period appears to have been too short for some countries to comfortably sustain the change upon exit. Exit is required and justified, as nongovernmental organizations the size of World Animal Protection cannot feasibly support rabies control in the medium to long term. The recommendation for future campaigns is to begin each engagement with a clear and transparent perspective of when the exit will occur, and what will need to be in place for this to be considered a successful exit by all stakeholders. This could potentially include fundraising support for alternative donors from the outset. Being transparent about exiting from the beginning of engagement can mitigate reputational risk from partners’ unmet expectations. Making the exit period at least equal in length to the full engagement period, if not longer, should be considered. Even after exit it is advisable to maintain a ‘watchful presence’, to protect investment in building a humane approach and be ready to mount a robust response if there appears to be a backwards step towards culling.

### 3.8. Preparation for Robust Response to Culling

During the period of ‘Red Collar’, there were examples of inhumane culling for rabies control occurring in Bali and Malaysia, apparently uninhibited by ‘Red Collar’ campaigning in protest. Future campaigns would benefit from developing pre-emptive strategies for robust response to sudden increases in inhumane practices such as culling. This may include garnering support from other influential stakeholders to be part of lobbying missions when required; although it should be noted that nongovernmental organizations often have the ability to speak as confrontational activists and agitators, where influential stakeholders such as intergovernmental organizations may not, being required to instead remain more diplomatic. Hence, the options both of working together and of working independently to combat inhumane practices should be explored. Such pre-emptive planning can be time consuming, but there is potential for high reputational benefits for responding quickly and effectively, versus the risk of high reputational loss if perceived to have not protested sufficiently.

## 4. Conclusions

The ‘Red Collar’ campaign had ambitious goals of driving governments to adopt humane and effective controls for dog-mediated rabies in five years. Evaluation of stakeholder perspectives suggested significant achievements in both policy and practice of humane control in target countries/districts, and when combined with ASEAN’s regional strategy, sufficient momentum appears to have been created to influence other nations to follow suit. However, it should be noted that credit for these changes cannot be attributed solely to the ‘Red Collar’ campaign, only contribution, as this campaign existed within a wider context of ongoing inherent government development and other campaigns.

Although there were achievements made within the campaign timeframe, there were other planned goals that were not met. The evaluation exposed various learning points that were developed into recommendations for improving campaign effectiveness in the future. These recommendations included continued emphasis on providing a consistent compassionate ‘voice’ for animals through campaigns, improved monitoring and evaluation of indicators relevant to stakeholders, and greater prominence of the role of capacity-building in theories of change where limits to government capacity are likely to be a barrier. The recommendations also called for closer evaluation of the motivations behind those negative practices targeted by ‘Red Collar’—in this case, culling of dogs for rabies control—and greater preparation for responding to renewed culling. The time required to achieve the stated goals and then exit, leaving behind a sustainable humane control approach to rabies, appears to have been underestimated. 

Objective data on campaign goals, most notably the numbers of dogs culled, was limited and hence, the evaluation had to depend almost solely on stakeholder interviews with corroboration of policy and practice from official documents, when available. Stakeholder interviews began with an introduction that offered anonymity to the interviewee and emphasized the learning focus of the evaluation, where all feedback would be treated as welcome input to building future campaign effectiveness. Although stakeholders appeared at ease in reporting both appreciation and criticisms of the campaign, and none asked for anonymity, it is still possible that stakeholders were influenced by factors such as interviewer bias and hopes for future funding or support from World Animal Protection. 

Campaigning organizations such as World Animal Protection can play a significant role in changing policy and practices that affect animal welfare, as evidenced by the ‘Red Collar’ campaign’s impact on increasing humane rabies control centred on mass dog vaccination as opposed to inhumane culling. The perspectives of many stakeholders gathered through this evaluation were used to identify several recommendations that have the potential to increase the impact of future campaigns for the benefit of dogs and the people they live amongst.

## Figures and Tables

**Table 1:** Breakdown of stakeholder type and geographical focus.

Stakeholder ^1^ Type	Geographical Focus					
	Global/Regional	Bangladesh	China	Indonesia	Philippines	Zanzibar
Government	-	5	2	1	2	7
IGO	5	1	-	4	-	-
World Animal Protection (WAP)	6	2	3	1	1	3
NGO (not WAP)	2	1	-	3	1	2
Medic	-	1	-	-	-	1
Community	-	-	-	-	-	4

^1^ Total no. of stakeholders exceeds 54—some stakeholders participated in more than one location. IGO: Intergovernmental organization; NGO: Nongovernmental organization.

## References

[1] Maclachlan N., Dubovi E.J. (2016). Fenner’s Veterinary Virology.

[2] Hampson K., Coudeville L., Lembo T., Sambo M., Kieffer A., Attlan M., Barrat J., Blanton J.D., Briggs D.J., Cleaveland S. (2015). Estimating the Global Burden of Endemic Canine Rabies. PLoS Negl. Trop. Dis..

[3] Fahrion A.S., Mikhailov A., Abela-Ridder B., Giacinti J., Harries J. (2016). Human rabies transmitted by dogs: Current status of global data, 2015. Wkly. Epidemiol. Rec..

[4] World Health Organization, World Organisation for Animal Health Background and conference objectives. Proceedings of the Global Elimination of Dog-Mediated Human Rabies: A Report of the Rabies Global Conference.

[5] Dürr S., Fahrion A.S., Knopf L., Taylor L.H. (2017). Editorial: Towards Elimination of Dog Mediated Human Rabies. Front. Vet. Sci..

[6] World Health Organization Rabies Factsheet. http://www.who.int/mediacentre/factsheets/fs099/en/.

[7] Hughes J., Macdonald D.W. (2013). A review of the interactions between free-roaming domestic dogs and wildlife. Biol. Conserv..

[8] Macpherson C.N.L., Meslin F.-X., Wandeler A.I., Macpherson C.N.L., Meslin F.-X., Wandeler A.I. (2013). Preface to the 2nd edition. Dogs, Zoonoses and Public Health.

[9] Miklosi A. (2007). Dog Behaviour, Evolution and Cognition.

[10] Morters M.K., Restif O., Hampson K., Cleaveland S., Wood J.L.N., Conlan A.J.K. (2013). Evidence-based control of canine rabies: A critical review of population density reduction. J. Anim. Ecol..

[11] Cleaveland S., Beyer H., Hampson K., Haydon D., Lankester F., Lembo T., Meslin F.-X., Morters M., Mtema Z., Sambo M. (2014). The changing landscape of rabies epidemiology and control. Onderstepoort J. Vet. Res..

[12] Hampson K., Dushoff J., Cleaveland S., Haydon D.T., Kaare M., Packer C., Dobson A. (2009). Transmission dynamics and prospects for the elimination of canine rabies. PLoS Biol..

[13] OIE World Organisation for Animal Health Chapter 7.7. Stray Dog Population Control. Terrestrial Animal Health Code. http://www.oie.int/fileadmin/Home/eng/Health_standards/tahc/current/chapitre_aw_stray_dog.pdf.

[14] Putra A.A.G., Hampson K., Girardi J., Hiby E., Knobel D., Mardiana I.W., Townsend S., Scott-Orr H. (2013). Response to a rabies epidemic, Bali, Indonesia, 2008–2011. Emerg. Infect. Dis..

[15] Lembo T., Hampson K., Kaare M.T., Ernest E., Knobel D., Kazwala R.R., Haydon D.T., Cleaveland S. (2010). The feasibility of canine rabies elimination in Africa: Dispelling doubts with data. PLoS Negl. Trop. Dis..

[16] World Health Organization Towards a Rabies-Free World as Unparalleled Global Initiative Gets Underway. http://www.who.int/neglected_diseases/news/WRD_2017_Press_release/en/.

[17] Tuckwell J. (2016). World Animal Protection. Auckland, New Zealand. Personal communication.

[18] Association of South East Asian Nations ASEAN Rabies Elimination Strategy. http://asean.org/storage/2017/02/ASEAN-Rabies-Elimination-Strategy.pdf.

[19] Green J., Thorogood N. (2014). Qualitative Methods for Health Research.

[20] Coffman J. Public Communication Campaign Evaluation: An environmental Scan of Challenges, Criticisms, Practice, and Opportunities. Harvard Family Research Project. http://citeseerx.ist.psu.edu/viewdoc/download?doi=10.1.1.575.7053&rep=rep1&type=pdf.

[21] The World Medical Association Inc Declaration of Helsinki: Ethical Principles for Medical Research Involving Human Subjects. https://www.wma.net/policies-post/wma-declaration-of-helsinki-ethical-principles-for-medical-research-involving-human-subjects/.

[22] Taplin D.H., Clark H., Collins E., Colby D.C. Theory of Change. Technical Papers: A Series of Papers to Support Development of Theories of Change Based on Practice in the Field. http://www.actknowledge.org/resources/documents/ToC-Tech-Papers.pdf.

[23] Food and Agriculture Organization Supporting National Efforts toward Zero Rabies in Viet Nam. http://www.fao.org/vietnam/news/detail-events/en/c/885168/.

[24] Institut Pasteur du Cambodge “Getting to Know about Rabies” Pasteur’s BOOK in Khmer to Promote the Fight against Rabies. http://www.pasteur-kh.org/2017/09/22/getting-to-know-about-rabies-a-book-in-khmer-to-promote-the-fight-against-rabies-published-sept25/.

[25] Cleaveland S., Kaare M., Tiringa P., Mlengeya T., Barrat J. (2003). A dog rabies vaccination campaign in rural Africa: Impact on the incidence of dog rabies and human dog-bite injuries. Vaccine.

[26] Townsend S.E., Sumantra I.P., Pudjiatmoko, Bagus G.N., Brum E., Cleaveland S., Crafter S., Dewi A.P.M., Dharma D.M.N., Dushoff J. (2013). Designing programs for eliminating canine rabies from islands: Bali, Indonesia as a case study. PLoS Negl. Trop. Dis..

[27] Belotto A., Leanes L.F., Schneider M.C., Tamayo H., Correa E. (2005). Overview of rabies in the Americas. Virus Res..

